# Can Humanism Be Infused Into Clinical Encounters in a Time-Constrained, Technology-Driven Era?

**DOI:** 10.7759/cureus.27836

**Published:** 2022-08-09

**Authors:** Ami Schattner

**Affiliations:** 1 Internal Medicine, Hebrew University Hadassah Medical School, Jerusalem, ISR; 2 Internal Medicine, Adelson School of Medicine, Ariel University, Ariel, ISR

**Keywords:** clinical encounter, communication, patient-physician relationship, quality of care, patient-centered care, empathy, humanism

## Abstract

A humanistic, personal, empathic, and patient-centered attitude towards the patient and family during the clinical encounter is often neglected in many settings. However, patients give it an utmost priority; moreover, the Institute of Medicine stressed it as a fundamental approach to improve the quality of care in the US, and the potential benefits accrued by its implementation are substantial. These benefits encompass patients (including increased satisfaction, trust, adherence, and ‘hard’ health outcomes), physicians (including rediscovering meaning and escaping burnout), and health care systems. Highlighting the quintessential value of humanism and patient-centeredness in the encounter, we discuss the cornerstones of adopting a 'personal' attitude that requires sincere friendly 'connecting' to the patient which can be accomplished with little loss of time, and their myriad advantages, to motivate clinicians to be more mindful of the patient and his or her circumstances.

## Editorial

“Technological advances do not equal ethical advances, and technology without ethics can beget harm instead of gains” - A. Varshizky

“We can only cure a small amount of human suffering. The rest of it needs to be healed” - R.N. Remen

"The secret of patient care is in caring for the patient". This succinct advice given by Francis Peabody in a talk to Harvard Medical School students a century ago is arguably even more valid today than when it was first conceived. Advances in medical science have dramatically improved outcomes; however, a patient, attentive, respectful, empathic, and patient-centered approach remains top patients’ priorities, possibly no less than the most sophisticated evidence-based treatment. Moreover, it is an acknowledged sine qua non of professionalism, high-quality medical care, and one of the patient’s elementary rights.

Unfortunately, some of these advances in modern medicine have inadvertently led to distancing and alienation between physicians and their patients. For example, in Peabody’s time, residents spent countless hours at their patients’ bedsides. Today, patient contact is restricted due to shortened patients’ lengths of stay and reliance on sophisticated imaging and laboratory tests rather than listening and examining the patient. Thus, as time-motion studies have demonstrated, residents spend only 9%-12% of their time in direct patient care vs. 40%-50% with the computer [1]. In primary care, circumstances are no better, since the meager 5.0-11.7 minutes per patient in the UK, and 9.2-21.07 minutes in the USA have not changed much in years, hardly sufficient for the older, often multicultural patient population characterized by increasing multimorbidity and polypharmacy [2]. Physicians’ escalating tasks and diagnostic or therapeutic options to choose from in a constantly time-constrained and supervised encounter add to their cognitive load, as does the frequent fragmentation of care [2,3]. Another example is the effect of these conditions on physicians. An appalling prevalence of burnout >50% is found among physicians worldwide, identified in every setting and specialty [2,3]. The COVID-19 pandemic likely added substantially to physicians’ burden. Burnout is a relatively new affliction, and as its components of emotional exhaustion and depersonalization suggest, it has a strong negative impact on physicians’ ability to relate to their patients and express humanism, not to mention increased medical errors, poorer quality of care, and diminishing patients’ trust and adherence associated with burnt out clinicians [4].

Let aside the beneficence inherent to the practice of medicine, and the basic patient’s right to autonomy and shared decision-making, humanistic care based on communication, empathy, and patient-centeredness is not only an obligatory requirement but affords numerous benefits not only for patients but for health care providers as well. Physicians who are sincerely interested in their patients, and provide a humanistic, personal, caring encounter as a routine, will thrive by the experience of the encounter and the different, often fascinating people and touching stories that unfold [5]. Patients readily distinguish caring physicians, and reward them with warm gratitude and trust that adds flavor and meaning to the physician through each clinical encounter, driving away fatigue and burnout. No less important, such an approach enables identifying and dealing effectively with ubiquitous contextual factors whose neglect (so-called "contextual error") may result in an inappropriate care plan. Crucial patients’ emotional problems, such as anxiety and depression, that may not be spoken of, but are highly potent and prevalent among ambulatory or hospitalized patients, are also considerably more likely to be detected and addressed. Even "biological" data will be more fully disclosed to physicians who are friendly and empathic. For patients, such an attitude fosters bonding, stronger patient-physician relationships, patient satisfaction, increased trust, increased adherence and enablement, and readiness to make an effort (often needed over extended periods) to adhere to medical recommendations. Relatively strong data support this virtuous cycle [6], also indicating decreased physicians’ risk of being sued. Data associating this kind of practice with improved ‘hard’ health outcomes is currently less robust and requires further study, though supported by several studies. With a subsequent decrease in tests, referrals, and prescriptions, system benefits are also likely, possibly including cost containment, less congestion, and retention of more satisfied physicians.

So, what constitutes humanistic care, and how can it be incorporated into the tight schedule of the encounter? Looking at quite sizable qualitative research, primarily based on patients’ preferences and interviews with clinicians renowned for their excellent patient relationships [5, 7-9], several recurring quintessential themes and techniques of sustained humanistic practice can be identified.

First, is the ingrained belief that knowledge, skills, evidence-based decisions, and patient-centered, empathic, humanistic care are two sides of the same coin, and only their combined application can result in healing. This knowledge, a belief in the many merits of patient-centeredness, and the forthcoming genuine gratitude of patients and families sustain the physician and nurture motivation. Second, the humanistic clinician adopts a tri-phasic attitude of effective empathy, a continuum of comprehension (cognitive-phase based on listening and understanding the patient’s narrative); compassion (an emotional response); and subsequently, an active commitment to support and do the best for this patient. The provider's compassion needs to be felt by the patient and followed by action. Third, disregarding all components of power and privilege and the patient’s vulnerable situation, the humanistic clinician truly sees himself or herself as a human being equal/similar to the patient in all but professional expertise. Respect and humility, being fully attentive and concentrated (and letting it be seen!), and sensing the situation from the patient’s point of view are directly derived from this attitude, and are major harbingers of humanistic care. Fourth, the humanistic clinician is curiosity-driven throughout. This applies to the clinical problem at hand but importantly also to the patient’s identity, circumstances, feelings, and to the physician’s own behavior (reflection is inward-directed curiosity). Curiosity infuses enthusiasm into the encounter. The physician likes the opportunity of meeting people, becoming privy to their stories, and trying to help them as best as possible. To start, one could initiate a personal, friendly exchange by responding to any patient cue (verbal/nonverbal) or asking something, using the patient’s name, country of origin, residence, occupation, or family as starting point. Fifth, the above-mentioned comprehension, compassion, sense of similarity, recognition of self-vulnerability and fallibility, humility, and often finding something to like and admire in the patient - yields being connected to the patient, a harbinger of reciprocal response and an antidote to burnout. To better achieve this, the caring clinician looks at the patient, acknowledges any accompanying persons, and openly demonstrates interest, sympathy, and commitment through explicit words and body language. To avoid focusing on the computer screen, a couple of minutes devoted before the encounter will get the patient’s essentials or the gist of previous visits. Performing physical examination (in addition to positive and many important negative findings) creates a unique connection. 

These themes that can be adopted and implemented by targeted education or self-taught, must be incorporated into the clinical encounter as a routine. Being an attitude that becomes part of the physician’s personality, it requires little practice and little time to implement. With these themes, we can quickly connect to the patient, get to know at least one personal attribute, and demonstrate sincere friendliness and commitment. The patient’s inevitable appreciation and gratitude will roll the ball of the dual relationship and the benefits are continuous and very substantial.

## References

[1] Block L, Habicht R, Wu AW (2013). In the wake of the 2003 and 2011 duty hours regulations, how do internal medicine interns spend their time?. J Gen Intern Med.

[2] Schattner A (2022). It's about time: redesigning consultation length in general practice. J R Soc Med.

[3] Harry E, Sinsky C, Dyrbye LN (2021). Physician task load and the risk of burnout among US physicians in a national survey. Jt Comm J Qual Patient Saf.

[4] Panagioti M, Geraghty K, Johnson J (2018). Association between physician burnout and patient safety, professionalism, and patient satisfaction: a systematic review and meta-analysis. JAMA Intern Med.

[5] Schattner A (2009). The silent dimension: expressing humanism in each medical encounter. Arch Intern Med.

[6] Little P, Everitt H, Williamson I (2001). Observational study of effect of patient centredness and positive approach on outcomes of general practice consultations. BMJ.

[7] Churchill LR, Schenck D (2008). Healing skills for medical practice. Ann Intern Med.

[8] Chou CM, Kellom K, Shea JA (2014). Attitudes and habits of highly humanistic physicians. Acad Med.

[9] Branch WT Jr, Weil AB, Gilligan MC (2017). How physicians draw satisfaction and overcome barriers in their practices: "It sustains me". Patient Educ Couns.

